# Transcriptomic Insights into the Effect of Melatonin in *Saccharomyces cerevisiae* in the Presence and Absence of Oxidative Stress

**DOI:** 10.3390/antiox9100947

**Published:** 2020-10-01

**Authors:** Mercè Sunyer-Figueres, Jennifer Vázquez, Albert Mas, María-Jesús Torija, Gemma Beltran

**Affiliations:** Departament de Bioquímica i Biotecnologia, Grup de Biotecnologia Enològica, Facultat d’Enologia, Universitat Rovira i Virgili, C/Marcel·lí Domingo, 1. 43007 Tarragona, Catalunya, Spain; merce.sunyer@urv.cat (M.S.-F.); jenvazk@gmail.com (J.V.); albert.mas@urv.cat (A.M.); gemma.beltran@urv.cat (G.B.)

**Keywords:** yeast, melatonin, oxidative stress, antioxidant, hydrogen peroxide, bioactive compound, hypoxia, mitochondria

## Abstract

Melatonin is a ubiquitous indolamine that plays important roles in various aspects of biological processes in mammals. In *Saccharomyces cerevisiae*, melatonin has been reported to exhibit antioxidant properties and to modulate the expression of some genes involved in endogenous defense systems. The aim of this study was to elucidate the role of supplemented melatonin at the transcriptional level in *S. cerevisiae* in the presence and absence of oxidative stress. This was achieved by exposing yeast cells pretreated with different melatonin concentrations to hydrogen peroxide and assessing the entry of melatonin into the cell and the yeast response at the transcriptional level (by microarray and qPCR analyses) and the physiological level (by analyzing changes in the lipid composition and mitochondrial activity). We found that exogenous melatonin crossed cellular membranes at nanomolar concentrations and modulated the expression of many genes, mainly downregulating the expression of mitochondrial genes in the absence of oxidative stress, triggering a hypoxia-like response, and upregulating them under stress, mainly the cytochrome complex and electron transport chain. Other categories that were enriched by the effect of melatonin were related to transport, antioxidant activity, signaling, and carbohydrate and lipid metabolism. The overall results suggest that melatonin is able to reprogram the cellular machinery to achieve tolerance to oxidative stress.

## 1. Introduction

Melatonin (*N*-acetyl-5-methoxytryptamine; Mel) is a versatile indolamine that is best known as a neurohormone in vertebrates. Since it was first discovered in the bovine pineal gland [1], it has been found in most living organisms [2]. In humans, melatonin has numerous physiological functions, such as regulating circadian rhythms and synchronizing the reproductive cycle, and it exhibits anti-aging, antioxidant and anti-inflammatory activities. It can even modulate a variety of neural, endocrine and immune functions [3,4].

Since Sprenger et al. [5] initially related melatonin production to *Saccharomyces cerevisiae*, several studies have reported the ability of yeasts to synthetize melatonin during alcoholic fermentation, with levels in wine ranging bewteen 0.3–1800 nM [6,7,8,9,10,11,12]. Little information on melatonin biosynthesis in yeast is available. Although a synthetic route similar to that described for vertebrates was initially reported [5], Muñiz-Calvo et al. [13] recently proposed a putative biosynthetic pathway including some steps described in plants, such as the synthesis of serotonin from tryptophan through tryptamine instead of 5-hydroxytryptophan. Another issue that remains to be deciphered is the physiological role of melatonin in yeasts. Recent studies have demonstrated a protective function of melatonin against oxidative stress and UV radiation [14,15,16] and indicated a hypothetical role as a signaling molecule [7]. The recent finding that melatonin interacts with glycolytic proteins during alcoholic fermentation reinforces the latter role, which seems to be linked to fermentative metabolism [17,18].

Oxidative stress is provoked by an imbalance in reactive oxygen species (ROS) resulting in ROS-mediated molecular and functional impairment and ultimately leading to cell death. To prevent these effects, cells employ antioxidant defense mechanisms in which enzymatic and nonenzymatic systems take part, neutralizing free radicals before they affect essential elements of the cell [19,20,21]. In yeast, exogenously applied melatonin has been reported to protect various biomolecules from damage caused by free radicals both directly, by scavenging ROS, and indirectly, by decreasing oxidized glutathione and activating genes that are involved in the oxidative stress response, such as glutathione/glutaredoxin, thioredoxin, catalase and superoxide dismutase genes [15,16]. ROS produce changes in the lipid bilayer composition resulting in lipid peroxidation, which is correlated with membrane disintegration and cell death. Recently, it has been shown that under oxidative stress, *Saccharomyces* takes advantage of melatonin supplementation by reducing the lipid peroxidation provoked by ROS, leading to an increase in total fatty acids and a higher proportion of unsaturated fatty acids, resulting in a higher tolerance to hydrogen peroxide (H_2_O_2_) [15].

In humans, the action of melatonin is mitochondrion targeted, as the electron transport chain (ETC), where higher ROS production occurs, is located in the mitochondria [22]. Therefore, melatonin not only reduces ROS damage by scavenging ROS and increasing antioxidant enzyme activities but also improves the efficiency of the ETC and ATP production (reviewed in Reiter et al. [23]). Zampol and Barros [24] revealed that the addition of melatonin to yeast cells improved respiration when the cells were challenged with a compound that induces oxidative stress (menadione), mainly by affecting complex IV activity, suggesting that melatonin may also exhibit mitochondrion-specific activity in yeast.

*S. cerevisiae* exhibits a number of inducible adaptive stress responses to oxidants such as H_2_O_2_, superoxide anions and lipid peroxidation products. The oxidative stress responses are regulated at the transcriptional level, and there is considerable overlap between them and the stress responses associated with other types of stresses (general stress response) [20,21]. Thus, the oxidative stress response is not mediated by an isolated linear metabolic or signaling pathway. Instead, cells are able to reprogram gene expression to optimize signal transduction for more efficient and effective adaptation, setting up a general stress response that encompasses a much larger stress signaling network and integrating information from many pathways [25,26,27]. The physiological changes induced in yeast by melatonin supplementation and the ways in which yeast cells respond to oxidative stress suggest that melatonin might be involved in multiple biological processes in yeast, and it is interesting to investigate whether melatonin acts as a signaling molecule that triggers a molecular and physiological response to cope with stress situations. Therefore, obtaining an accurate idea of how melatonin supplementation modulates gene expression is critical for understanding the signaling events that it triggers.

To gain insight into the antioxidant role and regulatory mechanism of melatonin in yeast, we evaluated the effect of melatonin on gene transcription after analyzing the ability of yeast to incorporate exogenous melatonin into the cell. For this purpose, we measured intracellular melatonin and performed a transcriptomic study in a commercial wine yeast strain of *S. cerevisiae*, in the presence and absence of both melatonin and oxidative stress. After analyzing the results, we validated the effect of melatonin on the lipid composition and mitochondria via physiological studies.

## 2. Materials and Methods

### 2.1. Yeast Strain and Experimental Conditions

The wine yeast QA23, a commercial strain of *S. cerevisiae* (Lallemand, Montreal, QC, Canada), was used in this study. Yeast precultures were prepared in YPD broth [2% (*w/v*) glucose, 2% (*w/v*) peptone and 1% (*w/v*) yeast extract (Panreac, Barcelona, Spain)], incubated at 28 °C with orbital shaking (120 rpm) for 24 h, and monitored by measuring the OD at 600 nm. Then, an initial population of 5 × 10^5^ cells/mL (OD_600nm_ 0.05) was inoculated into YPD broth (175 mL for melatonin quantification, 50 mL for transcriptomic assays, 300 mL for time course assays, 450 mL for lipid analysis and 60 mL for the quantification of mitochondria) with or without melatonin [TLC grade, purity ≥98%, Sigma-Aldrich (St. Louis, MO, USA)] supplementation (5 μM; Mel and Control conditions, respectively) and grown until the cells reached the initial exponential phase (OD_600nm_ 0.5–0.6) (designated time 0). Then, sublethal oxidative stress was induced with 2 mM H_2_O_2_ (MelH and H conditions, respectively), and samples were taken after 1 h by harvesting the cells via centrifugation at 28,672× *g* for 5 min at 4 °C. Then, the pellet was washed with Milli-Q water (Q-PODTM Advantage A10, Millipore, Burlington, MA, USA) and stored at −80 °C until use. The variations in this general procedure are specified below. The melatonin and H_2_O_2_ concentrations were chosen on the basis of our previous study in the QA23 strain [15]. A culture with 25 μM melatonin supplementation under stress conditions was added (25MelH) in the microarray analysis. Three biological replicates were employed in all assays.

### 2.2. Intracellular Melatonin Quantification

For intracellular melatonin quantification, 10^8^ cells were harvested from the samples at 0 h (just before stress induction) and 1 h after the stress induction by centrifugation at 4700× *g* for 15 min. Intracellular melatonin quantification was performed as previously described by Morcillo-Parra et al. [17]. In brief, melatonin was extracted by adapting the boiling buffered ethanol method described by Gonzalez et al. [28] and quantified by liquid chromatography mass spectrometry using a liquid chromatograph coupled to a triple quadrupole mass spectrometer (LC-MS/MS Agilent G6410A; Agilent Technologies Inc., Santa Clara, CA, USA). In parallel, 10^8^ cells were dried at 28 °C for 48 h to determine the dry weight of the samples, and the melatonin concentration was expressed as nM/mg dry weight.

### 2.3. Assessment of Global Gene Expression by Microarray Analysis

RNA was isolated from 10^7^ cells from each condition (Control, Mel, H, MelH, 25MelH) using a TRIzol^®^ Plus RNA Purification Kit from Ambion Life Technologies (Woburn, MA, USA), following the instructions of the manufacturer, but the chloroform step was repeated twice before transferring the upper phase containing the RNA to a fresh RNase-free tube. Furthermore, a DNAse (Qiagen, Barcelona, Spain) incubation step at 37 °C for 15 min was included to remove the remaining DNA. The RNA samples were quantified with a NanoDrop 1000 TM spectrophotometer (Thermo Scientific, Waltham, MA, USA), and their integrity was analyzed with an RNA 2100 Bioanalyzer (Agilent Technologies Inc.) using the RNA 6000 Nano kit and the Plant RNA Nano protocol in Agilent 2100 Expert software. Gene expression levels were assessed using a Yeast Gene Expression Microarray (8 × 15 K format) containing 6256 *S. cerevisiae* probes. Fifteen samples (three biological replicates of each condition) were analyzed. Each sample was labeled with Cy3 and hybridized through one-color microarray-based exon analysis (Low Input Quick Amp WT Labeling kit protocol version 2.0, Agilent Technologies) according to the manufacturer’s instructions.

#### Microarray Data Analysis

Agilent Scan Control version A.8.5.1 software was used to scan 3-μm-resolution slides using the Agilent G2565CA Microarray Scanner System with SureScan High-Resolution Technology. Feature extraction version 12.0.1.1 software (Agilent Technologies) was used for data extraction. Statistical transcriptomic analysis for identifying significant changes between the conditions was performed using Gene Spring GX Software v.13.1.1 from Agilent Technologies. The signal for each spot was normalized at the 75% percentile, and the moderated t-test with Benjamini-Hochberg multiple testing correction was used to designate the differentially expressed genes. The genes that were differentially expressed between two conditions (*p*-value < 0.05) were selected for further analysis: Venn diagrams were generated at the website http://bioinformatics.psb.ugent.be/webtools/Venn/, regulatory networks were generated with the PheNetic web tool [29] and the molecular functions, biological processes and cellular components were determined with Gene Ontology (GO) (CC-BY 4.0, [30]). The specific pathways involved in the differentially expressed genes were analyzed with the KEGG pathway mapping database [31], and significantly enriched pathways were determined with the DAVID tool [32,33]. The results of transcriptomic analysis were deposited in the Gene Expression Omnibus (GEO) repository, with GEO accession number: GSE154702 (https://www.ncbi.nlm.nih.gov/geo/query/acc.cgi?acc=GSE154702).

### 2.4. Gene Expression by qPCR Analysis

The expression of genes of interest was evaluated via qPCR before stress (0 h) and after exposure to stress (1, 4, 12 and 24 h for Control and Mel; 0.5, 1, 4, 12, 24 and 36 h for H and MelH). RNA was isolated from 10^7^ cells using the Universal RNA Purification Kit from EURx (Gdańsk, Poland) with some modifications: to improve cellular lysis, cells were resuspended with a mixture of 1% β-mercaptoethanol in lysis buffer [PureLink RNA mini kit (Invitrogen, Carlsbad, CA, USA)], added to a 2 mL screw-cap tube with 1 g of 0.5 mm-diameter glass beads, and lysed using a MBB-16 Mini-Beadbeater (BioSpec Products, Inc., Bartlesville, OK, USA) (5 cycles of 30 s in the Mini-Beadbeater + 30 s on ice). The suspension was centrifuged for 5 min at 21,952× *g* at 4 °C and transferred to a homogenization spin column. Thereafter, the protocol was followed as recommended by the manufacturer, with optional on-column DNase digestion with DNase I from EURx. The RNA samples were quantified with a NanoDrop 1000 TM spectrophotometer (Thermo Scientific) and stored at −80 °C. The obtained RNA, together with that obtained as described in Section 2.3, was converted to cDNA to evaluate the expression of genes of interest by qPCR (Table 1). The samples were prepared as follows: 12 µL of 320 ng/µL RNA, 1 µL of an Oligo (dT)20 primer (Invitrogen), 1 µL of dNTPs (10 mM), 4 µL of buffer, 1 µL of DTT and 1 µL of SuperScript IV Reverse Transcriptase (Invitrogen), and amplification was performed according to the instructions of the manufacturer using a 2700 Thermal Cycler (Applied Biosystems, Waltham, MA, USA) and stored at −20 °C.

The primers for each evaluated gene (Table 1) were designed using Primer Express software (Primer Express 3.0 Applied Biosystems), and we employed the genes *ACT1, HEM2* and *TAF10* as endogenic controls. All primers were supplied by Invitrogen, and a standard curve was performed for each pair of primers. qPCR was performed using 2 µL of cDNA diluted 10-fold, 0.4 µL of each primer, 0.08 µL of ROX [SYBR Premix Ex Taq II (TaKaRa Bio Inc, Shiga, Japan)], 10 µL of SYBR Green (SYBR Premix Ex Taq II) and 7.12 µL of sterile Milli-Q water. Amplification was conducted using a QuantStudio5 Real Time PCR system (Thermo Fisher Scientific) as follows: one cycle of 95 °C for 1 min and 40 cycles of 95 °C for 5 s and 60 °C for 35 s, followed by a dissociation step. Relative gene expression was calculated with Thermo Fisher Cloud software (Thermo Fisher Scientific) using the 2^−ΔΔCt^ formula, where Ct is defined as the cycle at which fluorescence is determined to be statically significantly above background; ΔCt is the difference between the Ct of the gene of interest and the mean value for the endogenous controls, and ΔΔCt is the difference between the ΔCt values under the different conditions (see figure legends for relative expression details). Two biological replicates were analyzed under each condition.

### 2.5. Analysis of Sterols, Fatty Acids and Phospholipids

Yeast cell homogenates were obtained, and the protein content was quantified as described by Vázquez et al. [36]. Total lipids were extracted from cell fractions corresponding to 0.5 mg, 1 mg or 3 mg of total cell protein for sterols, fatty acid (FA) or phospholipid (PL) assays, respectively, according to the method described by Folch et al. [37]. The composition of each lipid was determined as described by Vázquez et al. [36]. In brief, the composition of individual sterols was determined via gas-liquid chromatography-mass spectrometry (GC-MS) after the alkaline hydrolysis of the lipid extracts as described by Quail and Kelly [38]; to this aim a Hewlett-Packard 5690 Gas Chromatograph (Agilent Technologies) equipped with an HP 5972 mass selective detector using a capillary column (HP 5-MS; 30 m × 0.25 mm i.d. × 0.25 µm film thickness) was employed. The FA composition was determined by gas-liquid chromatography (GLC) using a Hewlett-Packard 6890 gas chromatograph [39], and the PLs were first separated by two-dimensional thin layer chromatography (TLC) [40]. Later, individual PLs were scraped off the plate and quantified by estimating the amount of phosphates [41]. Two biological replicates were performed.

### 2.6. Quantification of Mitochondria

Mitochondria were stained with the fluorescent dye MitoTracker Green (Thermo Fisher Scientific). Cells were grown in YPD with or without melatonin (5 and 50 µM) until the cells reached the initial exponential phase, and oxidative stress was then induced (with 0, 2 or 5 mM H_2_O_2_) for 1 h. A total of 10^7^ cells were harvested and directly (without a freezing step) resuspended in 1 mL of PBS (phosphate-buffered saline) with a final concentration of 100 nM MitoTracker Green and incubated for 10 min at room temperature protected from light. Fluorescence was measured via flow cytometry, and data acquisition was performed with FloMax software (Quantum Analysis GmbH, Münster, Germany) and processed with WinMDI 2.9 software (Joseph Trotter, Salk Institute for Biological Studies, La Jolla, CA, USA). The mean fluorescence index (MFI) was calculated according to Boettiger et al. [42]: [(geometric mean (Gmean) of the positive fluorescence)-(Gmean of the control)]/(Gmean of the control).

### 2.7. Data Analysis

Data obtained from the intracellular melatonin quantification, lipid content, mitochondrial quantification and qPCR analyses were subjected to analysis of variance (ANOVA) and Tukey’s post hoc test using GraphPad Prism 7 (GraphPad Software, San Diego, CA, USA) and XLSTAT 2019 software (NY, USA). All the results were considered statistically significant at a *p*-value < 0.05. To merge standard deviations, the program “combine means and SDs into one group program” of the StatsToDo website (https://www.statstodo.com/index.php) was used.

## 3. Results and Discussion

In previous studies, we analyzed the antioxidant effects of exogenous melatonin on *S. cerevisiae* at the physiological level [15,16]. Our data showed slight increases in ROS and oxidized glutathione under melatonin supplementation when no stress was induced. In contrast, when cells were under oxidative stress, melatonin activated some genes involved in the yeast antioxidant defense systems, thus reducing ROS accumulation and increasing cellular viability [16]. In this study, we wanted to investigate the effect of melatonin on the yeast global transcriptomic response to gain insight into its antioxidant role and regulatory mechanisms.

### 3.1. Differential Gene Expression Profiling

**Figure 1:**
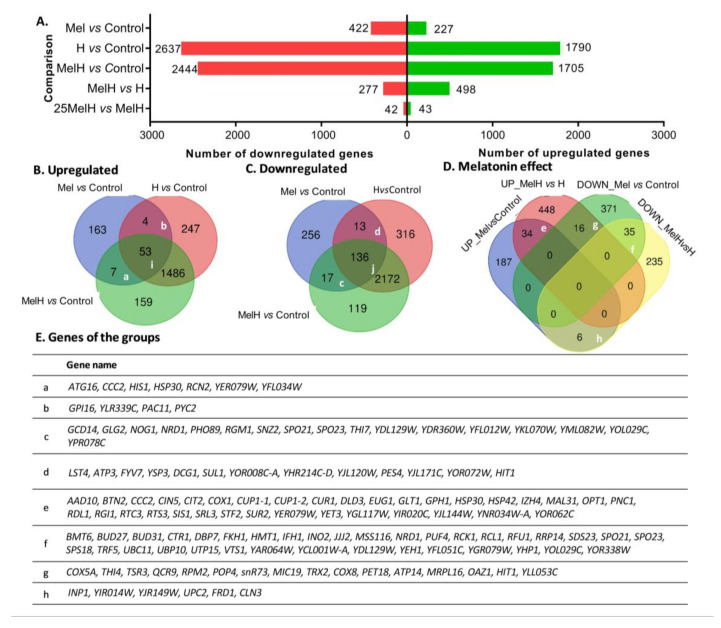
Distribution of differentially expressed genes in yeast cells grown with and without melatonin (5 μM or 25 μM), in stressed (2 mM of H_2_O_2_) and in unstressed conditions (Control, Mel, MelH, 25MelH and H). (**A**) The number of differentially expressed genes between different conditions (fold change −1 ≤ FC ≥ 1; *p*-value < 0.05). (**B**,**C**) Venn diagram showing the number of common genes found among the Mel vs. Control, MelH vs. Control and H vs. Control comparisons (up- (**B**) and down (**C**) regulated genes). (**D**) Venn diagram showing the number of common genes found among the up- and downregulated genes identified in the Mel vs. Control and MelH vs. H comparisons. (**E**) List of genes in some groups indicated in (**B**–**D**). The list of genes and the GO enrichment analysis of all the groups is shown in Table S1.

To obtain an overview of the gene expression profile associated with melatonin supplementation in *S. cerevisiae*, a comparative transcriptomic analysis was performed between cells that were grown with and without melatonin supplementation (5 μM) and with and without oxidative stress exposure (2 mM H_2_O_2_) (Control, Mel, H and MelH conditions). The overall results obtained from the transcriptomic analysis can be found in Datasets S1–S10: differentially expressed genes (*p*-value < 0.05) are listed in Datasets S1–S5, and the results for all genes are provided in Datasets S6–S10 and at the GEO repository (https://www.ncbi.nlm.nih.gov/geo/query/acc.cgi?acc=GSE154702). The numbers of genes showing significant changes in global gene expression (with a *p*-value < 0.05) under each condition are represented in Figure 1. A total of 649 genes were differentially expressed under exogenous melatonin treatment (Mel vs. Control), mainly downregulated (422 genes), while oxidative stress resulted in the altered expression of 4427 genes (Figure 1A; H vs. Control). Among these genes, 3658 were affected by stress independent of the presence of melatonin (common to H vs. Control and MelH vs. Control but not Mel vs. Control) (Figure 1B,C). However, melatonin clearly altered the gene expression profile of stressed cells, with 775 genes being differentially expressed (MelH vs. H), most of which were upregulated (498 genes), in contrast with the downregulation effect of melatonin on expression in nonstressed cells (Figure 1A). Furthermore, the effect of the melatonin concentration under oxidative stress was evaluated by testing a higher concentration (25 μM melatonin, 25MelH). Its effect was weak, with only 85 genes exhibiting altered expression due to the higher concentration of melatonin (Figure 1A, 25MelH vs. MelH, Datasets S5 and S10). Thus, 5 µM melatonin was sufficient to modulate gene regulation in yeast cells.

Our results showed 189 genes were differentially expressed under all three conditions (Mel, H, MelH) in comparison with the control (Figure 1B(i), Figure 1C(j)), most of which were downregulated and were involved in mitochondrial function (Table S1). Additionally, 24 genes were regulated by melatonin regardless of the stress treatment (common in Mel vs. Control and MelH vs. Control, Figure 1B(a), Figure 1C(c)). Most of them were also downregulated and were mainly involved in nutrient regulation (*NRD1*, *SNZ2* and *RGM1*) or gene transcription (*GCD14*, *NOG1* and *NRD1*) (Figure 1E(c)). On the other hand, when we focused on the effect of melatonin in the cell, under stressed and nonstressed conditions, we found 69 genes that were commonly up- or downregulated by melatonin in both conditions (Figure 1D, groups e and f) and 22 genes that were regulated in opposite direction in stressed or nonstressed cells (Figure 1D, groups g and h). The upregulated genes were involved in the response to ROS (including both metallothioneins, *CUP1-1* and *CUP1-2*) and water deprivation, protein folding, oxidation-reduction process (*COX1*), copper binding (metallothioneins and *CCC2*), zinc homeostasis (*IZH4*) and the transport of maltose (*MAL31*), oligopeptides (*OPT1*) and ions (*HSP30*) (Figure 1D,E, group e, Table S1). The genes downregulated by melatonin were mainly related to transcription and the regulation of gene expression (Figure 1D,E, group f, Table S1). On the other hand, several mitochondrial genes presented opposite behavior in relation to melatonin treatment; i.e., they were downregulated in nonstressed cells and upregulated in stressed cells, mainly being involved in the electron transfer chain and ATP synthesis, including genes related to cytochrome c oxidase (*COX5A, COX8*) and reductase activity (*QCR9*), ATP synthase (*ATP14*), and mitochondrial organization and stability. In this group, there were also genes related to thiamine metabolic processes and RNA processing (Figure 1D,E, group g, Table S1).

### 3.2. Classification of Differentially Expressed Genes into Functional Categories

**Figure 2:**
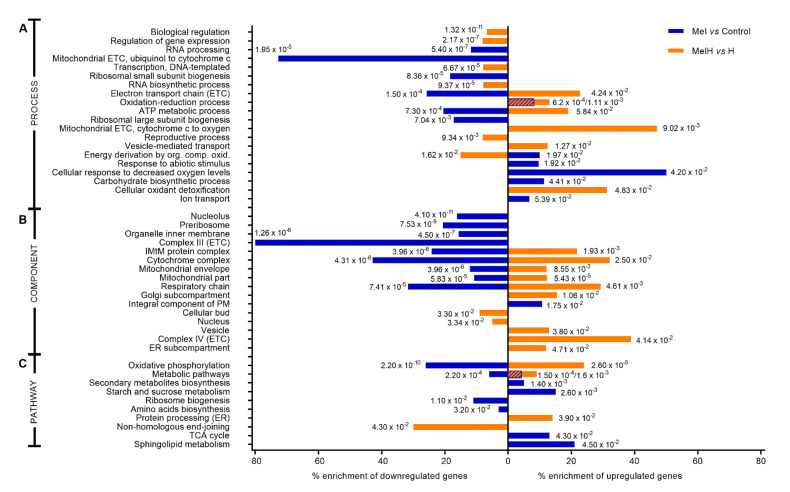
Categories enriched by differentially expressed genes that were up- (values to the left) and down- (values to the right) regulated (1 ≤ fold change ≥1; *p*-value < 0.05) among nonstressed (blue) and stressed (orange) (2 mM H_2_O_2_) cells in the absence or presence of melatonin (5 μM). Percentages of enrichment are calculated as the ratio of the number of up- or downregulated genes in relation to the total number of genes involved in each molecular function or pathway in *S. cerevisiae*. The numbers correspond to the *p*-values. When two *p*-values are presented, the left one corresponds to Mel vs. Control and the right to MelH vs. H. (**A**) Biological process function enrichment from Gene Ontology (GO) analysis, (**B**) cellular components enriched according to GO analysis, (**C**) pathway enrichment analyzed with the DAVID tool. Abbreviations: ETC, electron transport chain; ER, endoplasmic reticulum; TCA, citrate cycle, IMtM, inner mitochondrial membrane.

To understand the role of melatonin supplementation in yeast, GO enrichment analysis was performed to determine which molecular functions, biological processes, cellular components and pathways were overrepresented among the differentially expressed genes under the different conditions. In this work, we mainly focus on the results obtained under conditions with the addition of melatonin with or without stress. The overall results obtained from the GO enrichment analysis with the genes included in each category can be found in the Supplementary Materials (Datasets S11–S19). Figure 2 and Table 2 show the main biological annotations significantly associated with genes that were upregulated or downregulated by melatonin.

In the presence of melatonin (Mel vs. Control), the oxidation-reduction process and the responses to abiotic stimuli and chemicals were the biological processes that were most significantly represented among the upregulated genes, followed by the response to decreased oxygen levels, which showed the highest enrichment (50%), including 4 upregulated genes (*YLR256W, FRD1, MGA2, UPC2*) (Figure 2A and Dataset S13). Additionally, molecular functions related to transport activity and transmembrane transport were significantly overexpressed in the presence of melatonin, with 29 genes upregulated being found in this category (Table 2). Several pathways related to metabolism, especially that of carbohydrates (sucrose and TCA cycle) and sphingolipids, were also enriched (Figure 2C). In the case of downregulated genes, the most affected categories were related to mitochondrial electron transport and oxidative phosphorylation, with 50% of the cytochrome complex being downregulated, mostly represented by genes associated with complex III (80% enriched) and cytochrome c reductase activity therein (Figure 2, Table 2). Moreover, categories related to ribosome biogenesis and the structures in which it takes place (preribosomes and nucleolus) were also highly enriched among the downregulated genes, together with categories related to RNA processing (Table 2, Figure 2).

Categories related to mitochondria were also highly enriched in the MelH vs. H comparison, as observed in the Mel vs. Control comparison; however, in this case, these categories were enriched in upregulated genes, such as complex IV and associated cytochrome c oxidase activity, which was the most enriched component (40%), resulting in 32% enrichment of the cytochrome complex (Figure 2, Table 2). Categories related to antioxidant function and detoxification were also significantly overrepresented (Figure 2A, Table 2). The downregulated genes were enriched mainly in categories related to gene transcription, reproduction, and the regulation of these processes (Table 2, Figure 2).

Some of the GO categories and genes regulated by melatonin with or without stress will be discussed in more detail in the following sections. In the case of higher concentrations of melatonin (25MelH vs. MelH), although different gene expression profiles were observed, no significant differences were detected in the GO enrichment analysis.

### 3.3. Effect of Melatonin on Transport and Membrane Composition

Our transcriptomic study showed that the melatonin-upregulated genes were involved in transmembrane transporter activity in nonstressed cells, including transporters of ions [*HSP30* (also upregulated by melatonin in stressed cells)*, CCC2, VMA3, YHK8, FET3, FET4, VMA11*], amino acids (*DIP5, GAP1, AGP1, MUP3*), oligopeptides (*OPT1, OPT2, PTR2*) and urea (*DUR3*), among others (Table 2). Moreover, pathways related to lipid metabolism and peroxisomes were clearly altered in the presence of melatonin in both nonstressed and stressed cells. The melatonin-upregulated genes were involved in fatty acid (FA) elongation (13% and 38% enrichment in nonstressed and stressed cells, respectively) and the biosynthesis of unsaturated fatty acids (UFAs) (10% and 30%) and sphingolipids (21% and 7%, Figure 2) (*SUR2,* independent of the presence of oxidative stress, and *LAC1* and ISC1 in nonstressed cells) (Table S2). Genes related to peroxisome and β-oxidation were also affected (13%, 15%) by melatonin and were both up- and downregulated.

In vertebrates, several of the functions of melatonin are mediated by its membrane receptors [43], but others are receptor-independent, such as antioxidant activity, for which melatonin is required to penetrate the cell and enter intracellular compartments [44]. In humans, melatonin is suggested to cross membranes by passive diffusion and through transporters of glucose [45] or oligopeptides [46,47]. Moreover, as the membrane is the first barrier that separates the cell from the environment, it is one of the main targets of oxidative stress, which alters its lipidic composition. Yeast cells can sense oxidative stress and change their membrane composition to achieve tolerance against stress, and this response varies among yeast strains and species [36]. Indeed, the diversity in the membrane composition in different yeast species and strains seems to lead to different levels of tolerance against oxidative stress [36]. Moreover, melatonin modulates the FA composition and peroxisome proliferation in stressed and nonstressed cells, suggesting that it could influence the lipidic composition of cell membranes to achieve tolerance to oxidative stress [15].

Therefore, because melatonin modulated the expression of several genes involved in membrane transport and lipid metabolism, it was of interest to determine whether yeast cells are able to incorporate exogenous melatonin, whether this incorporation is altered by oxidative stress and whether the presence of melatonin in the medium affects the yeast membrane composition, either to prepare the cell to tolerate oxidative stress or to deploy a response that could lead to better tolerance against this stress.

#### 3.3.1. Intracellular Melatonin

**Figure 3:**
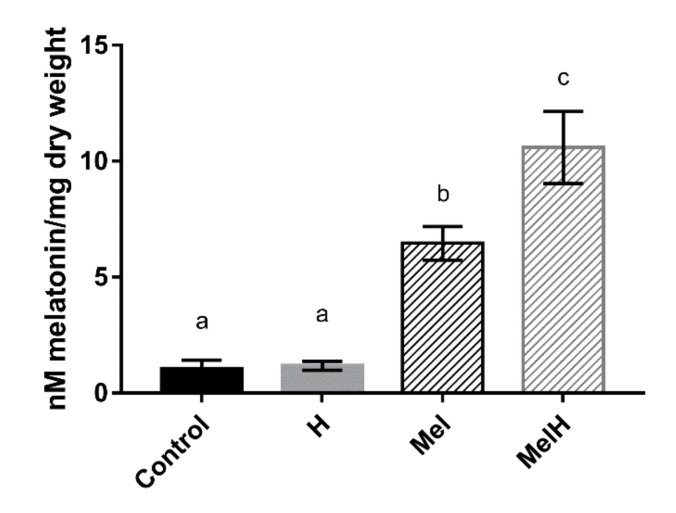
Intracellular melatonin quantification in cells untreated and treated with 5 μM of exogenous melatonin, before (Control and Mel) and after (H and MelH) being exposed to oxidative stress with 2 mM of H_2_O_2_. Error bars represent standard deviation, letters show statistical differences with *p*-value < 0.05.

To evaluate whether the QA23 strain was able to incorporate exogenous melatonin into the cell, intracellular melatonin was quantified under our four different conditions (Control, Mel, H and MelH) (Figure 3). Intracellular melatonin levels significantly increased when the cells were grown with melatonin supplementation independent of oxidative stress, indicating that *S. cerevisiae* (QA23 strain) was able to take up exogenous melatonin at nanomolar concentrations. Similar results have been previously reported in yeast [14] and mammals [48,49]. Our results also showed that the highest levels were reached in stressed cells that were previously grown in the presence of melatonin (Figure 3). The higher melatonin levels in stressed cells may be due to the changes induced by H_2_O_2_ in both plasma membrane permeability and the gradient thereof, which might promote changes in cellular transport [50] favoring the entry of melatonin into the cell. Another possible route through which melatonin may enter the cell is potentially through some membrane transporters, such as those for urea or polyamines (*DUR3*) (as they present a similar structure to melatonin), the oligopeptide transporter *OPT1*, or the maltose permease *MAL31*, the last two of which were upregulated by melatonin in presence and absence of oxidative stress (Figure 1E). Huo et al. [46] recently described the possible involvement of *PEPT1/2* oligopeptide transporters in the uptake of melatonin in mammalian cells, and a new line of evidence has shown that glucose transporters are linked to melatonin uptake in human cells [45,47]. Indeed, our results showed that some genes encoding carbohydrate and carbon source transporters were upregulated by the presence of melatonin in stressed cells (*HXT5, HXT6, HUT1, JEN1*, Dataset S4), which could also be related to melatonin internalization. Nevertheless, further studies should be performed to elucidate the possible role of these transporters in melatonin uptake by yeast cells.

#### 3.3.2. Physiological Changes in the Lipid Composition

**Figure 4:**
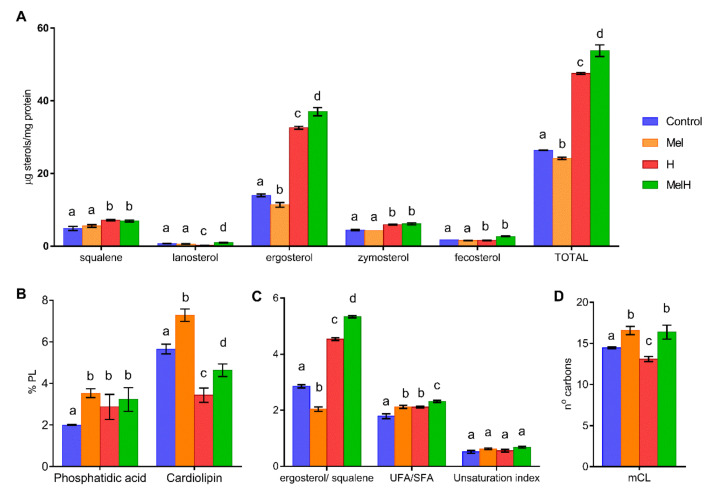
Lipid composition of nonstressed and stressed yeast cells with 2 mM of H_2_O_2_, grown with and without 5 μM of melatonin (Control, Mel, H, MelH). (**A**) Sterols. (**B**) Phospholipids. (**C**) UFA/SFA ratio (from Vázquez et al. [15]) and unsaturation index defined as follows: [(% C16:1 + % C18:1) + 2 (% C18:2) + 3 (% C18:3)]/100. (**D**) Medium-chain-length of FA (mCL, from Vázquez et al. [15]). Letters indicate significant differences between lipid concentrations (*p*-value < 0.05).

To test whether the abovementioned transcriptomic modifications resulted in changes in the cell lipid composition, we measured the sterol and PL contents of the yeast cells under the four different conditions (Control, Mel, MelH and H, Figure 4). In a previous study, we measured the changes in the fatty acid composition under these four conditions [15].

In nonstressed cells, melatonin supplementation resulted in lower total sterol levels than in the control condition, primarily because of a lower ergosterol content (Figure 4A), leading to a lower ergosterol/squalene ratio (Figure 4C). The opposite situation was found under oxidative stress, with higher levels of total sterols, mainly due to increased ergosterol, leading to a higher ergosterol/squalene ratio (Figure 4A,C). A study that compared the membrane composition of different yeast strains showed that the most H_2_O_2_-tolerant strains exhibited a low sterol content before stress and an increased ergosterol/squalene ratio after oxidative stress [36], behavior that we observe with melatonin supplementation in the present study. Moreover, an increase in total sterols has been related to higher tolerance to H_2_O_2_ and other stress conditions [51]. This suggests that one of the mechanisms by which melatonin confers resistance against H_2_O_2_ could be alteration of the ergosterol composition. The observed physiological behavior seems opposite to the pattern of the regulation of genes involved in sterol biosynthesis and transport, as these genes were upregulated in nonstressed cells (*ARE1, DAN1, IZH4, FHN1, HES1, UPC2*) and downregulated in stressed cells (*ARE2, ERG11, SWHI1, UPC2*). This could be explained by the regulatory role of Upc2p, a transcription factor that activates genes involved in sterol biosynthesis and transport and anaerobic genes and is activated under sterol depletion and anaerobic conditions (reviewed by Joshua and Höfken [52]). Therefore, its targets would be downregulated in the presence of high sterol concentrations (in stressed cells, melatonin downregulated *ERG11*) and upregulated in the presence of low sterol concentrations (in non-stressed cells, melatonin upregulated *FHN1*, *ARE1*, *HES1* and particularly *DAN1*, a cell wall mannoprotein and sterol transporter). Thus, it seems that the increase in the ergosterol content in the presence of melatonin could be produced during the first hour of oxidative stress, and genetic regulation could be a response to the sterol concentration. This increase in the ergosterol content could be driven by either posttranscriptional enzyme activation or higher oxygen availability, which is required for ergosterol biosynthesis [53].

On the other hand, the changes in FAs produced by melatonin were independent of stress, with higher oleic and palmitoleic acid contents [15] leading to a higher total FA level, a higher UFA/SFA ratio and an increased percentage of medium chain length (mCL) FAs in Mel and MelH conditions (Figure 4C,D). This increase was correlated with the induction of the genes involved in the FA synthesis and elongation pathways (*TES1* in the absence of oxidative stress and *TSC13*, *PHS1*, *ETR1* in the presence of oxidative stress), among which the thioesterase gene *TES1*, which is involved in the last step of the synthesis of several UFAs (such as oleic acid), and the *TSC13* and *PHS1* genes, which are involved in long-chain FA elongation, were the most affected by melatonin treatment. Moreover, high UFA/SFA ratios have been related to higher tolerance to H_2_O_2_ [54]. In fact, yeast strains with higher H_2_O_2_ tolerance showed higher UFA/SFA ratios before and after stress [36], with increases in palmitoleic and oleic acid levels being observed after stress, as observed under melatonin treatment. These results suggest that melatonin changes in the membrane FA composition improve the tolerance to oxidative stress. Genes related to β-oxidation and peroxisomes were mostly upregulated by melatonin under oxidative stress in this study (*POX1*, *PXA1*, *PEX19*), which suggests that melatonin could increase the β-oxidation of FAs inside peroxisomes. These results are consistent with the higher FA content observed under melatonin treatment, but they are not consistent with the increased peroxisome proliferation observed in nonstressed cells and the decreased peroxisome proliferation observed in stressed cells [15].

Finally, the only observed changes in the PL content were related to phosphatidic acid (PA) and cardiolipin (CL). Melatonin only increased PA levels in the absence of stress, and although the CL level decreased in the presence of oxidative stress, it was higher under both conditions involving melatonin supplementation (Figure 4B). Vázquez et al. [36] observed that all investigated strains showed a decrease in CL after stress exposure, but the strains that were most tolerant to H_2_O_2_ displayed higher CL values, as observed under melatonin treatment. In humans, melatonin prevents the peroxidation of cardiolipin to protect mitochondria from damage provoked by aging [22]. However, these results were not correlated with gene expression data, which revealed that in stressed cells, melatonin upregulated some of the genes involved in the biosynthesis of phosphatidylcholine (*CPT1*) and sphingolipids (*SUR2*), which are related to stress tolerance.

### 3.4. Response to Oxidative Stress

Our results showed that 32% of the genes involved in antioxidant activity were upregulated by melatonin in stressed cells (Table 2), including glutaredoxins (*GRX1*, *GRX2*), sulfiredoxin (*SRX1*) and four peroxidases (*DOT5, HYR1, GPX1* and *CTT1*), together with other genes with oxidoreductase activity, such as thioredoxins (*TRX1*, *TRX2*) and methionine-S-sulfoxide reductase (*MXR1*) (Table 2). These results were consistent with our previous studies [16], in which we observed that genes such as *GPX1*, *GRX2*, *TRX2* and *CTT1* were also induced by melatonin in *S. cerevisiae* stressed cells. Instead, in non-stressed cells, melatonin upregulated the cytoplasmic thioredoxin peroxidase *TSA2* but downregulated its paralog *TSA1* and the thioredoxin *TRX2*, mitochondrial superoxide dismutase *SOD2* and glutathione peroxidase *GPX2* genes (Dataset S1). Therefore, melatonin seems to downregulate the antioxidant response in the absence of oxidative stress. Other studies point in the same direction, as they have also shown that the expression of *GPX1*, *SOD2*, *GPX3* and *CTA1* was downregulated in the presence of melatonin [14,16].

To confirm our transcriptomic data, some of the genes related to stress tolerance showing higher expression ratios in MelH vs. H condition were verified by qPCR analysis using the same conditions as in the transcriptomic assay: *SRX1*, which encodes a sulfiredoxin that contributes to protection against oxidative stress [55,56]; *ADY2*, which encodes a carboxylic acid transporter of the plasma membrane implicated in acetic acid tolerance [57]; and *MGA1*, a stress-responsive gene that regulates Cis1p (explained in Section 3.6). In spite of the results of the Mel vs. Control comparison, which were nonsignificant, the MelH vs. H results showed tendencies that supported the results of the microarray analysis (Table S4).

Moreover, independent of the application of stress, melatonin upregulated the copper transporter *CCC2* and both metallothioneins (*CUP1-1* and *CUP1-2*), which have antioxidant and superoxide dismutase activity, and are involved in the detoxification of metal ions and removal of superoxide radicals, and downregulated the copper transporters *CTR1* and *CTR3* (the latter only in nonstressed cells) (Figure 1, Table 2). *FET3*, which contributes to resistance to copper toxicity, was upregulated by melatonin in the absence of oxidative stress (Table 2, Dataset S1). In a previous study, we observed that melatonin upregulated other metal-related antioxidants, such as *SOD1* [16]. Therefore, our results seem to indicate that melatonin activates a response against the toxic effects of metal exposure in *S. cerevisiae*, as reported in human cells [3,58], even without the presence of metals, as a mechanism for better enduring further stresses.

### 3.5. Effect of Melatonin on the Mitochondria

Many of the genes regulated by melatonin were located in mitochondria (89 genes in Mel vs. Control and 107 in MelH vs. H), mainly in the respiratory chain complexes and mitochondrial envelope (Figure 2, Table 3); these results suggested that the mitochondria could be the biological target of melatonin, as observed in humans [59]. However, the effects of melatonin on mitochondrial genes were opposite in stressed and nonstressed conditions, since melatonin mainly downregulated mitochondrial genes in nonstressed cells but upregulated these genes in stressed cells (Table 3).

In non-stressed cells, most of the repressed genes were related to the ETC, particularly to cytochrome c reductase [complex III, including two catalytic (*RIP1, CYT1*) and six additional subunits], as well as complexes IV, V and other ETC-related elements, such as NADH dehydrogenase, a cytochrome c isoform and enzymes involved in the process of obtaining the heme groups of ETC complexes. Melatonin also downregulated most of the genes related to other mitochondrial functions, such as protection, morphology, transport, mitoribosome and intergenomic signaling between the nucleus and mitochondria. However, some genes were also upregulated, such as *SHH4* of complex II of the ETC, a respiratory supercomplex factor, and genes involved in the TCA cycle and heme synthesis (Table 3). On the other hand, in stressed cells, the induced mitochondrial genes were mainly related to cytochrome c oxidase (complex IV), including not only genes that encode the central units (*COX1-3*) but also genes related to assembly (such as *ATP20*), stability, regulation or enzyme activity. Melatonin also upregulated genes associated with other ETC complexes as well as a respiratory supercomplex factor or a cytochrome c isoform and genes related to mitochondrial functions such as protection, morphology, mitoribosomes, intergenomic signaling or the TCA cycle. However, in the transport, translation and metabolism categories, there were both up- and downregulated genes.

#### 3.5.1. Gene Validation

**Figure 5:**
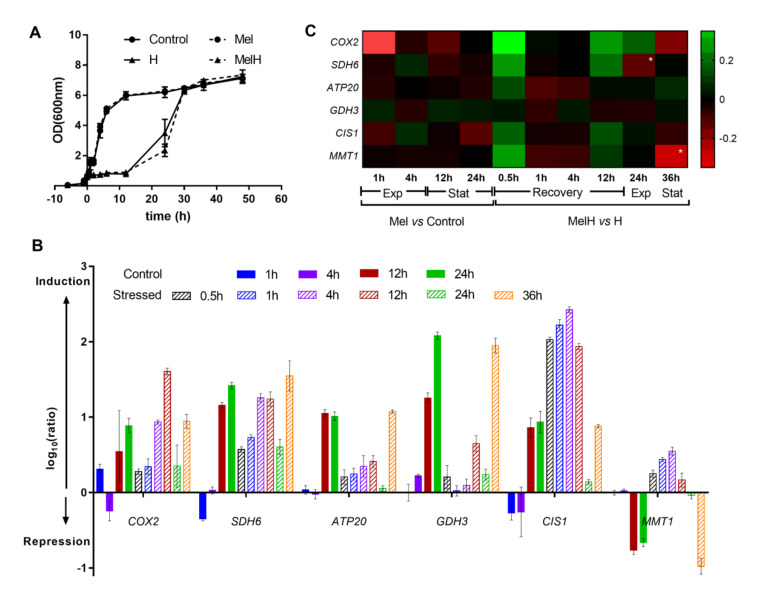
Expression of six selected genes over a time course. (**A**) Kinetics of yeast growth according to OD_600nm_ values. The cells were incubated with or without melatonin (5 µM) for 6 h, and two conditions (Mel, MelH) were subjected to 2 mM H_2_O_2_ treatment (0 h). (**B**) Expression of each gene of interest at different time points in the nonstressed and stressed conditions (Control, H), expressed as log_10_(ratio). The ratio represents the 2^−ΔΔCt^ value corrected to the 2^−ΔΔCt^ value of the Control at 0 h. (**C**) Heat map of the effect of melatonin on the expression of the six genes during the time course analysis in nonstressed and stressed cells. The color corresponds to the log_10_ of the ratio. The ratio represents the 2^−ΔΔCt^ value for the condition with melatonin corrected to the 2^−ΔΔCt^ value for the condition without melatonin. Genes are presented in rows, and the time points under both conditions are presented in columns. Exp, exponential phase; stat, stationary phase. High-intensity colors refer to values >0.35 (green) or < −0.35 (red) * significance level of < 0.05. Error bars represent the standard deviation, and the statistical analysis of B and C is presented in Table S3.

As mitochondria were the most enriched component, to confirm our transcriptomic data, the expression of some mitochondrion-related genes showing higher expression ratios in the MelH vs. H comparison were verified through qPCR analysis. We chose genes related to different mitochondrial functions, such as genes involved in the respiratory chain (*COX2, SDH6, ATP20*), structure (*ATP20*), mitochondria protection (*GDH3, CIS1*) and transport (*MMT1*) (Figure 5).

Gene expression was analyzed at the same sampling points used in the microarray assays and at different growth times under the four conditions (Control, Mel, H, MelH), and the results are shown in Figure 5. Most of the selected genes were upregulated either by entry into the stationary phase or by the response to oxidative stress exposure (which inhibited cell growth for 12 h, Figure 5A), following a general environmental stress response profile [60]. In nonstressed cells (Control condition), almost no changes in the expression of these genes were observed during the exponential phase (1 and 4 h) (Figure 5A,B), and only some of the genes were slightly downregulated (*SDH6, CIS1*) or upregulated (*GDH3*). However, the expression of all the genes (except *MMT1*) increased significantly in the stationary phase (12 and 24 h) (Figure 5A,B). Exposure to oxidative stress upregulated the expression of all genes, and these changes were significant for some of them at 1 h and for all the rest, except for *GDH3*, at 4 h (Table S3); their expression then decreased during the exponential phase (24 h), reaching the same levels observed before the stress in some cases, and significantly increased at the stationary phase (36 h) (Figure 5B). An exception was observed for *MMT1*, whose expression decreased in the stationary phase, as observed in the control condition.

The effect of melatonin on the expression of these genes in stressed and nonstressed cells is presented in Figure 5C. In nonstressed cells (Mel vs. Control), melatonin slightly decreased the expression of most of the genes in the early exponential phase (1 h), but practically no changes were observed in the exponential and stationary phases (Figure 5C). In stressed cells (MelH vs. H), melatonin induced most of the genes immediately after stress was applied (0.5 h), indicating an additive effect to the stress, after which the expression of most genes was slightly lower in MelH than in H conditions (1–4 h), followed by an increase just before entering the exponential phase (12 h). Thereafter, no important changes were observed in the exponential and stationary phases, except in *SDH6* and *MMT1*, respectively, whose expression was lower under MelH conditions (Figure 5C). Overall, the qPCR results supported those of the microarray analysis (Figure 5C, Table S4).

#### 3.5.2. Physiological Effect of Melatonin in Mitochondria

**Figure 6:**
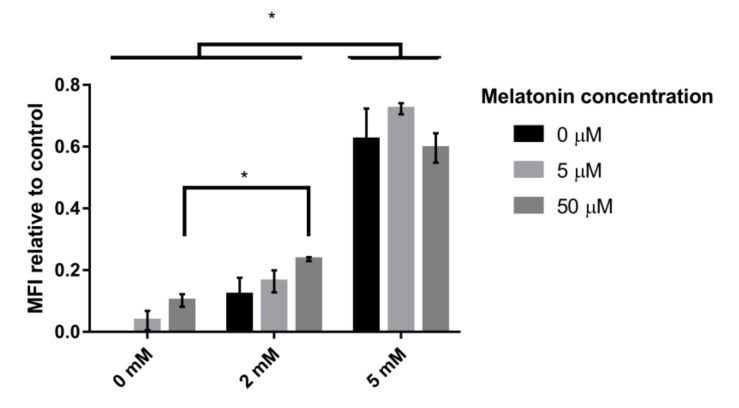
Effect of H_2_O_2_ and melatonin on the number of mitochondria per cell. The values are expressed as the mean fluorescence intensity (MFI) of the different conditions compared to the control. The cells were incubated with different melatonin concentrations (0 µM, 5 µM and 50 µM) and then subjected to treatment with different H_2_O_2_ concentrations (0 mM, 2 mM and 5 mM). Error bars represent the standard deviation, and * indicates statistically significant differences between media (*p* < 0.05).

To determine whether the high impact of melatonin on mitochondrial gene expression revealed physiological effects such as an increase in the concentration or activity of mitochondria, we analyzed the number of mitochondria per cell using MitoTracker Green, a dye that accumulates in the active mitochondria of living cells, regardless of the mitochondrial membrane potential. When the cells were subjected to oxidative stress (2 mM H_2_O_2_) for 1 h, fluorescence in the cells increased, indicating a greater number of mitochondria per cell. This increase was greatest, and the difference was statistically significant in the presence of a high concentration of H_2_O_2_ (5 mM) (Figure 6). Moreover, in nonstressed cells or stressed cells in the presence of a low concentration of the oxidative compound (2 mM), the presence of melatonin increased the number of mitochondria, which became even higher when the melatonin concentration was increased (50 µM). However, when the stress was increased (5 mM), the presence of melatonin did not affect the number of mitochondria per cell, which was already quite high due to the intensification of oxidative stress (Figure 6). These tendencies were also observed in the presence of 10 mM H_2_O_2_ (data not shown).

The observation that melatonin affects complexes in different ways depending on the presence or absence of oxidative stress is supported by the validation analysis of two genes necessary for the growth during respiratory metabolism, *COX2* and *ATP20*: melatonin upregulated these genes after oxidative stress and downregulated them in non-stressed cells. *COX2* encodes a central subunit of Complex IV necessary to assemble a functional complex, and *ATP20* encodes a subunit of Complex V that produces a functional association with Complex IV, which is essential to achieve maximum levels of Complex IV activity [61].

This, together with the increase in complex IV activity observed by Zampol and Barros [24], shows similar behavior to that of melatonin in human cells, in which melatonin increases the activity and gene expression of Complex IV [62,63]. In mammals, one of the main roles of melatonin is the maintenance of the respiratory chain flux, mainly by increasing the activity of Complexes I and IV [64,65]. Since *S. cerevisiae* lacks Complex I, it seems that the action of melatonin could be focused on Complex IV, possibly due to the important role of Complex IV in ETC regulation. Defects in Complex IV increase ROS production, so this complex seems to be an indicator of the cell oxidative capacity and thus to be pivotal in ETC regulation [66,67,68]. On the other hand, Complex III was the most affected by melatonin in non-stressed cells, in which most of the genes related to this complex were repressed. In mammalian cells, despite the clear antioxidant function of melatonin, several works have reported that melatonin *per se* can act as a prooxidant to induce ROS generation in several cell lines [69,70,71], an effect that has also been suggested in yeast cells [16]. Recently, it was revealed that the ROS generation induced in mammalian cells by melatonin at micromolar concentrations occurs via Complex III [72]. Moreover, Hardeland [73] noted in a review that Complex III activity has been found to be unchanged or upregulated by melatonin in different studies in different cell types. However, in yeast, melatonin seems to repress the expression of this complex; therefore, the effect of melatonin in this complex must be further studied.

Melatonin differentially regulates the expression of genes involved in mitochondrial protection. *GDH3*, which encodes a protein involved in the glutathione system and ROS reduction in the stationary phase, is upregulated in non-stressed cells and downregulated in stressed cells (1 and 12 h) by melatonin. In contrast, melatonin downregulated the expression of *CIS1* in nonstressed cells but upregulated its expression after stress exposure (0.5 and 12 h) (Figure 5). Cis1p, a mitochondrial protein whose biological role is unknown in yeast, has been recently reported to participate in the mitochondrial import surveillance mechanism, alleviating protein import stress [74]. Mitochondrial import stress has been associated with an increase in the mitochondrial mass and with mitochondrial stress conditions, such as high levels of ROS [74]. Therefore, the observed induction of *CIS1* in conditions that resulted in an increase in the mitochondrial content per cell (H and MelH, Figure 5 and Figure 6) supported the hypothesis that this induction could be a response to maintain active mitochondrial import and, thus, mitochondrial function. Moreover, *CIS1* is induced by the transcription factor *PDR3*, which also mediates a multidrug resistance (MDR) response, and in our work, the conditions of stress and melatonin treatment (in stressed cells) also upregulated *PDR1,* another transcription factor related to MDR (reviewed in Kolaczkowska and Goffeau [75]). Therefore, one of the actions of melatonin could be the triggering of the MDR response.

Our results indicated that melatonin plays a very important role in mitochondria, suggesting that it could be the biological target of melatonin, as observed in humans [59], because in both organisms, mitochondria are the site of higher production of ROS/RNS [22] and therefore the site where the reported antioxidant effect of melatonin is most needed. Nevertheless, melatonin seems to play roles other than counteracting the effects of oxidative stress (as seen in mammalian cells [76]) because while the effect of melatonin is focused on the ETC, this stress mainly affects the mitoribosomes and the inner membrane (Datasets S11–S19), suggesting that the targets of melatonin in mitochondria could be different from those observed in association with oxidative stress. This is supported by the observation that melatonin affects different complexes and genes in different ways depending on the presence or absence of oxidative stress.

### 3.6. Effect of Melatonin on Cell Signaling

**Figure 7:**
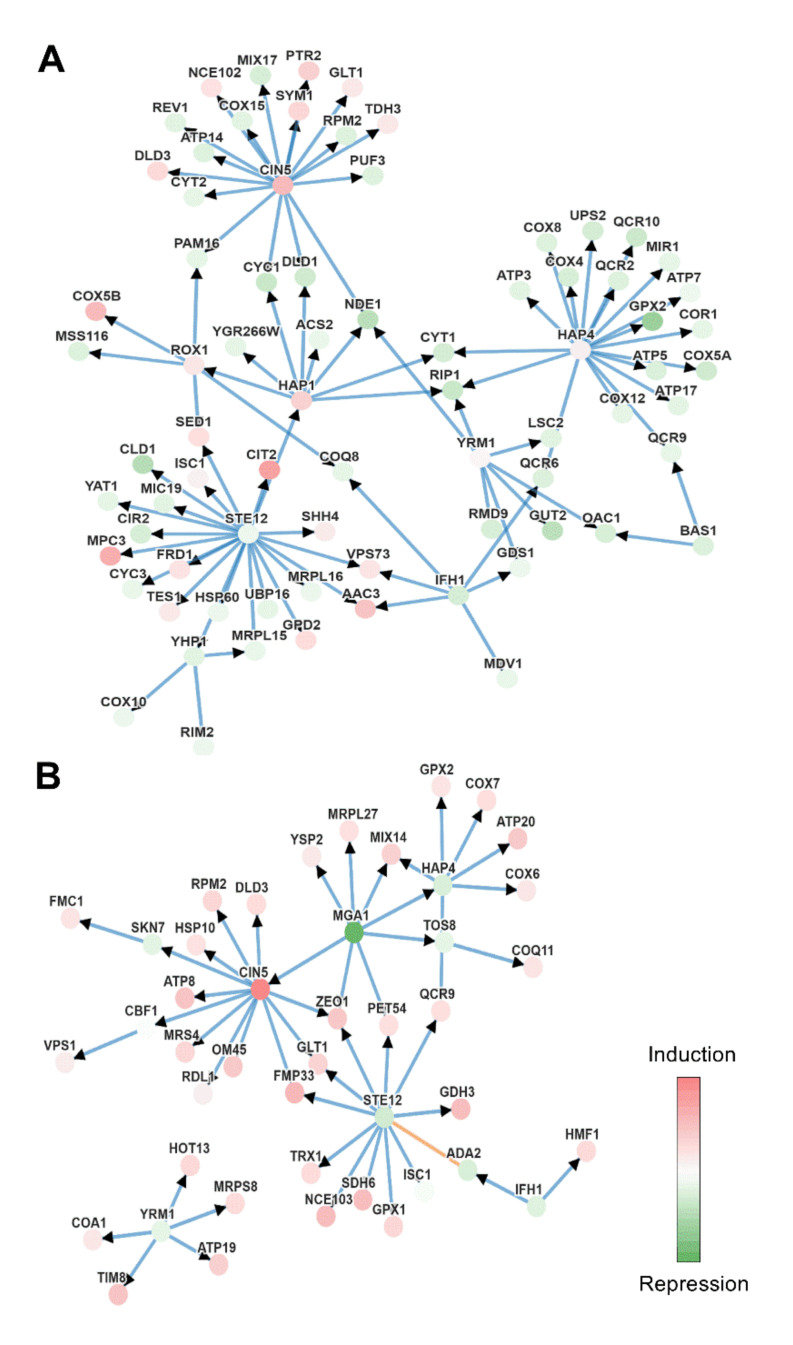
Genetic interactions given by Phenetics using mitochondrial genes with −1 < FC < 1, *p*-value < 0.05. Set of genes used in each comparation: (**A**) Mel vs. Control. (**B**) MelH vs. H. Nodes represent molecular entities, and the intensity of its color represents the level of differential expression, green being under-expression and red over-expression. The edges indicate the interaction between the genes (blue indicates protein-DNA and orange protein-protein), and the arrows the direction of the interaction.

The transcription factors involved in the different conditions were determined using PheNetic, a web tool that uses publicly available interactomics data to create networks and reveal possible relevant regulators. An interaction network was created using all the genes that were found to be differentially expressed (−1 < FC > 1, *p*-value < 0.05) in the Mel vs. Control and MelH vs. H comparisons to identify the relevant regulators of the effects of melatonin (Figure S1A,B). As melatonin seemed to have a great effect on mitochondria, another network was created using only the mitochondrial genes that were differentially expressed in these conditions (Figure 7A,B).

The analysis highlighted Cin5p and Ste12p as the central transcription factors among the genes regulated by melatonin in stressed and non-stressed cells (Figure S1). *CIN5*, which was upregulated by melatonin in all conditions, is a member of the Yap family and is upregulated after the application of an external stimulus such as oxidative or osmotic stress (reviewed in Rodrigues-Pousada et al. [77]). Cin5p induces genes upregulated by melatonin in stressed cells (such as *SRX1*, *SET6* or *HSP30*) and in non-stressed cells (such as *HSP42*, *ISF1*, *GSY1*, *PIG2*, *HSP30* or *GAC1*) [78,79,80]. Both *CIN5* and *ADY2,* which were upregulated by melatonin in stressed cells in our study (Figure S1B, Table S4, Dataset S4), are known to be activated by Msn2/4p stress-responsive transcription factors [58,81]. This suggests that melatonin could increase the response to oxidative stress in stressed cells, indicating an additive effect to the stress, which supports the results of Section 3.4 regarding the response to oxidative stress.

Ste12p, Mga1p (a node in MelH vs. H, Figure S1B) and Gat3p (a node in Mel vs. Control, Figure S1A) regulate filamentation and activate genes related to pseudohyphal/invasive growth [82,83]. These transcription factors seemed to be downregulated by melatonin, which suggests that melatonin could modulate filamentous growth in *S. cerevisiae*, as reported for some aromatic alcohols (such as phenylethanol and tryptophol) [84,85] produced (similarly to melatonin) through aromatic amino acid metabolism. 

When we focused only on mitochondrial genes, the same transcription factors were identified together with some additional transcription factors, such as Hap4p and Yrm1p in both stressed and nonstressed cells and Hap1p and Rox1p only in the Mel vs. Control comparison (Figure 7). Hap1p, Hap4p and Rox1p are heme-dependent transcriptional regulators. In the presence of heme, the Hap2/3/4/5 complex (in which Hap4p provides the activation domain) and Hap1p activate and upregulate genes required for aerobic growth, and Hap1p is also responsible for activating Rox1p, a repressor of hypoxic-related genes.

Therefore, in low-oxygen conditions (hypoxic growth), Rox1p is not expressed, and hypoxia-related genes are upregulated (reviewed by Siso et al. [86]). In our study, melatonin downregulated genes related to aerobic conditions in non-stressed cells (mitochondrial or nonmitochondrial) that are targets of Hap1p (*NDE1*, *CYC1* but also *CIR2* and *CLD1*), Hap2/3/4/5 complex (*COX5A*, *COX4* but also *INH1*) or both factors (*CYT1*, *RIP1*) (Figure 7, Dataset S1) [79,87].

Under the same conditions, melatonin also upregulated several hypoxia-related genes that are targets of Rox1p, including three of the most upregulated genes (*DAN1*, *HEM13* and *ANB1*), several mitochondrial genes (*COX5B*, *AAC3* or *RCF2*) and other nonmitochondrial genes with high fold-changes (FCs) (*FET4*, *ARE1*, *GAC1*, *SUR2*, *FET3* and *LAC1*) (Figure 2 and Figure 7C, Dataset S1) [79,80]. All of these results suggest that melatonin could activate a hypoxic response in yeast cells, despite the slight upregulation of the transcription factors *HAP1*, *HAP4* and *ROX1* (which was only significant for *HAP1*) (Dataset S1). Indeed, Hap1p is known to be regulated by its interaction with heme at the protein level but not at the transcriptional level (reviewed at Siso et al. [86]).

Other results obtained also supported the notion that melatonin could trigger a hypoxic response in nonstressed cells, such as the enrichment of the “response to decreased oxygen levels” among the upregulated genes in the Mel vs. Control comparison (Figure 2, Dataset S13). Among these genes, we found *UPC2*, a sterol regulatory element that controls the expression of an anaerobic sterol transport system. The regulation of sterol import is tightly connected to anaerobic conditions, as it is specific to anaerobic growth [53,88]. Under anaerobic conditions, Upc2p is upregulated and induces the expression of the *DAN/TIR* genes, a group of eight cell wall mannoproteins that are expressed under anaerobic conditions [89]. Indeed, our results showed the upregulation of four genes of this complex, *DAN1, DAN4, TIR1* and *TIR4*, which are associated with the enriched “structural constituent of the cell wall” molecular function category (Table 2). *DAN1*, a well-known hypoxic-related gene, was the gene showing the highest FC in the Mel vs. Control comparison (Dataset S1). Some of our results agreed with those of Bendjiali et al. [90], which describes a response specific to hypoxia.

Indeed, it has been reported that hypoxia induces the formation of foci containing glycolytic enzymes, which increases the incorporation of carbon into pyruvate and oxaloacetate [91]. Recently, melatonin has been shown to bind a complex of glycolytic enzymes [17], and this binding would be related to yeast fermentative capacity [18]. Several glycolytic genes found in this complex, such as *PYC2* and *TDH3*, were upregulated by melatonin in non-stressed cells (Figure 1B,E, Dataset S1). Therefore, it seems that melatonin could trigger a hypoxia-like response in non-stressed *S. cerevisiae* cells regulated by the Hap complex, which could increase their fermentation performance. Nevertheless, more studies are needed to examine this hypothesis.

## 4. Conclusions

This study is the first to reveal the yeast transcriptional response in the presence of exogenous melatonin. *S. cerevisiae* was able to incorporate exogenous melatonin, which affected genome-wide gene expression levels upon entering the cell. In the absence of stress, melatonin exposure appears to activate genes related to transport, oxidoreductase activity and hypoxia and to downregulate genes related to mitochondria and the ETC (mainly Complex III), triggering a hypoxia-like response, which could enhance fermentation performance. Indeed, some heme-dependent transcriptional regulators (Hap1p, Hap4p, Rox1p), together with the stress response factor Cin5p, seem to play a crucial role in the effect of melatonin at the transcriptional level. These changes in gene expression could also prepare yeast cells for coping with additional possible stresses such as oxidative stress. It is well established that under environmental stress conditions, the cellular machinery of yeast is reprogrammed to achieve better adaptation to stress, affecting not only genes involved in antioxidant defenses but also genes involved in lipid metabolism and reproduction. In this context, melatonin might enhance energetic efficiency and signal transduction, conferring higher H_2_O_2_ tolerance to *S. cerevisiae*. Under oxidative stress, melatonin upregulates genes related to antioxidant activity, cellular detoxification, oxidoreductase activity and the respiratory chain (mainly Complex IV), inducing transcriptional and physiological changes in yeast mitochondria. Thus, in stressed cells, melatonin supplementation seems to contribute to the stabilization of the mitochondrial electron chain, as observed in humans. However, as also shown in humans (reviewed in Cardinali and Vigo [22]) melatonin can play other roles in the yeast mitochondria: it increases the cardiolipin concentration and acts as a mitochondrion-targeted antioxidant at both physiological and transcriptional levels, activating genes related to mitochondrial function and maintenance.

## Figures and Tables

**Table 1:** Primers used in this study for the analysis of gene expression by qPCR.

Gene Name	Nucleotide Sequence of Forward Primer (5′ to 3′)	Nucleotide Sequence of Reverse Primer (5′ to 3′)
*ACT1*	TGGATTCCGGTGATGGTGTT	CGGCCAAATCGATTCTCAA
*ADY2*	TTTCAGCCTTCGCGTTGAC	CTTGCGCTCTCGCATTGA
*ATP20*	GGGTCTTCAACCACCAACTGTT	GGCTCTGCTTATAAAGGTTCGAAT
*CIS1*	CCCATCGGGTTAGTTTCAAAAA	GACATGCTACCCACTCTGCAATAG
*COX2*	TCGTTGTAACAGCTGCTGATGTT	CCAGGAGTAGCATCAACTTTAATACCT
*GDH3*	CACCGGGTTCGGCTTAGTT	GCCGTTTGTTGCATAATCGA
*HEM2*	TTCCGCTATTCATCTCCGATAATCCAG	ACAGACATCGCAAATAATATACAGTTCAGG
*MGA1*	ATGGGCAGTCCCGTCCATTACT	TCGCATCATGTTCACCGTGGGT
*MMT1*	GCGTTGTTTGCGGATGCTA	GCAAAGTCAACAAGTCAGAAACCA
*SDH6*	ACTTCACCACCATTGAACACTTGT	GGGTGTGAAAAGGTGGCAATT
*SRX1*	CCTGTGTTGGATCCTCAA	GGCATAATATAGCGTCTGTC
*TAF10*	ATATTCCAGGATCAGGTCTTCCGTAGC	GTAGTCTTCTCATTCTGTTGATGTTGTTGTTG

Primer design was performed using Primer Express software (Primer Express 3.0 Applied Biosystems). The primer ACT1 was previously reported by Beltran et al. [34], and those for HEM2 and TAF10 were previously reported by Teste et al. [35].

**Table 2:** Molecular function enrichment according to the Gene Ontology (GO) analysis of differentially expressed genes that were up- or downregulated (fold change ≥ 1; *p*-value < 0.05) among nonstressed and stressed (2 mM H_2_O_2_) cells in the absence or presence of melatonin (5 μM) (Mel vs. Control, MelH vs. H). Percentages of enrichment are calculated as the ratio of the number of up- or downregulated genes in relation to the total genes involved in each molecular function in *S. cerevisiae*.

GO Term Molecular Function (*p*-Value)	% Enrichment	Gene Names
**Mel vs. Control**		
**Upregulated (227 genes)**		
Transporter activity (0.00174) Trasmembrane transporter activity (0.01011)	7.21	*OPT2, MEP1, SWH1, VMA11, YHK8, AGP1, COX5B, FET4,* *AZR1, LAM5, FET3, PTR2, VMA3, HES1, GAP1, OPT1,* *FCY21, MPC3, VPS73, STE6, CCC2, YMR279C, AAC3,* *MAL31, DIP5, COX1, MUP3, HSP30, DUR3*
Oxireductase activity (0.01995)	6.96	*YGL039W, HEM14, CUP1-2, COX1, SUR2, MDH2, GPD2,* *RNR3, HEM13, TSA2, CUP1-1, FRD1, AAD10, EUG1,* *MPO1, DLD3, HMG2, FET3, YJR149W, YGL185C, GLT1,* *TDH3, COX5B, SHH4*
L-serine ammonia-lyase activity (0.02)	75.00	*YIL168W, CHA1, YIL167W*
Structural constituent of cell wall (0.053)	16.28	*TIR4, TIR1, SED1, PIR5, DAN1, YBR067C, DAN4*
**Downregulated (422 genes)**		
Ubiquinol-cytochrome-c-reductase activity (2.11 × 10^−5^)	77.78	*COR1, QCR10, QCR8, QCR2, QCR9, QCR6, YEL024W*
Electron transfer activity (2.57 × 10^−5^)	28.07	*COX8, COX12, CYT1, QCR9, QCR6, COX4, CYB5,* *YEL024W, COX5A, QCR2, COR1, CIR2, DRE2, QCR8,* *CYC1, QCR10,*
Inorganic molecular entity transmembrane transporter (0.09017)	11.72	*MPH3, MMP1, AQR1, ATP3, ATP14, YBR294W, VBA3,* *ATP7, OAC1, YLL053C, MIR1, PHO89, COX12, COX8, HNM1, TAT2, YPR124W, PHO84, FEX2, ATP17, CTR3, CTR1, COX4, CTP1, KCH1, PRM6, ATP5, YBR219C,* *COX5A*
**MelH vs. H**		
**Upregulated (498 genes)**		
Oxireductase activity (2.24 × 10^−6^)	15.74	*GDH3, COX8, QCR9, AYR1, ALD3, SDH4, COX5A, GLT1,* *POX1, COX1, CUP1-2, HYR1, YKL071W, CUP1-1, MPD1,* *TRX2, CYC7, GPX1, HOM6, FRE6, COX6, DLD3, TRX1,* *COX3, MXR1, YKL107W, FRE3, COX7, COQ11, GTO1,* *CTT1, TSC13, GIS1, CIR1, YPR127W, YCR102C, DOT5,* *TPA1, GAL80, SER33, SUR2, SRX1, PRM4, HBN1, COX2,* *QCR7, AAD10, FDH1, ETR1, GRX2, GRX1, EUG1, HFD1,* *YJR096W*
Electron transfer activity (0.00877)	24.56	*CIR1, GRX2, GRX1, COX7, COX8, QCR9, CYC7, PRM4,* *COX6, COX5A, QCR7, COX2, COX3, COX1*
Antioxidant activity (0.01019)	32.26	*CUP1-2, DOT5, HYR1, GRX2, CUP1-1, GRX1, GPX1, SRX1, GTO1, CTT1*
Cytochrome-c oxidase activity (0.02878)	41.18	*COX1, COX3, COX7, COX2, COX5A, COX6, COX8*
Oxidoreductase activity acting on a sulfur group of donors (0.08819)	25.64	*TRX1, GTO1, SRX1, PRM4, EUG1, GRX2, MPD1, TRX2,* *GRX1, MXR1*
**Downregulated (277 genes)**		
Helicase activity (0.0165)	14.16	*YRF1-6, YRF1-7, DBP1, ARP5, YRF1-5, YHL050C, MSS116, MPH1, DBP7, YLL067C, YEL077C, YRF1-8, DHH1, SNF2,* *DBP3, YKU80*

**Table 3:** List of mitochondrial genes regulated by melatonin in nonstressed cells (Mel vs. Control) and stressed cells (MelH vs. H). The fold change and *p*-values are provided in Datasets S1 and S4. They are classified into different categories, and the genes that were upregulated and downregulated in each condition are listed.

Category/Element	Mel vs. Control		MelH vs. H	
	Upregulated	Downregulated	Upregulated	Downregulated
Complex II of ETC	*SHH4*		*SDH4, SDH8, SDH6*	
Complex III of ETC		*RIP1, CYT1, COR1, QCR2, QCR6, QCR9, QCR10, QCR8*	*QCR7, QCR9*	
Complex IV of ETC	*COX1, COX5B*	*COX5A, COX10, COX15, COX4, COX8, COX12*	*COX1, COX2, COX3, COX5A, COX6, COX7, COX8, COX17*	
Complex V of ETC		*ATP3, ATP5, ATP7, ATP17, ATP14*	*ATP8, ATP14, ATP15, ATP18, ATP19, ATP20*	
Other genes related to ETC	*RCF2*	*CYC1, CYC3, CYT2, COX10, COX15, NDE1*	*RCF1, CYC7, COQ10, COA1, CMC2*	*ALD5, COQ1*
Mitochondrial protection	*HSP78*	*SOD2, HSP60, GPX2*	*CIS1/YLR246C, GDH3, GPX1, TRX1, HYR1*	
Mitochondrial import		*PAM16, PAM18*	*TOM7, TIM8, HOT13, PAM17*	*XDJ1, MSK1, YJR045C*
Mitochondria morphology		*MIC19, MIC12, FMP30, MDV1*	*MIC19, MIC26, GET1*	
Mitoribosome	*PPE1*	*MRPL7, MRPL16, MRPL51, MEPL15, MRPL19, MRPL6, MRPS28, MRP21*	*MRPL16, MRPL44, MRPL32, MRPL27, YNL122C, MRP2, MRP17, MRPS5, RSM18, MRPS8*	*GEP3, MRP4*
Mitochondrial translation		*RPO41, RMD9, RPM2, MSS116, MTG2*	*CBP6, RPM2, RTC6, PET54*	*MSS116, MSS51, PET111, PET9*
Intergenomic signaling		*COR1, QCR2, QCR6, COX5A, COX8, ATP3, ATP5, ATP7, ATP14, ATP17, BNA4, MIR1, SOD2*	*COX6, COX5A, COX7, COX8, ATP14, ATP20, QCR7, SDH4*	
Mitochondrial transporters	*MPC3, AAC3*	*MIR1, CTP1, RIM2, YAT1, OAC1*	*HSP10, HXT6, OM14, MRS4, CRC1*	*MMT1, MDL1, GNP1, ATM1, YHM2*
TCA cycle	*MDH2, CIT2, SHH4*	*LSC2*	*CIT2, SDH4, SDH8, SDH6*	
Metabolism (other)	*HEM13, HEM14, CHA1, ICT1, SYM1*	*GUT2, ARG8, EHT1, CLD1, FOL1, UPS2, DLD1*	*YHR208W, AYR1, NCE103, CPT1, HFD1, ETR1*	*MAE1, HEM1*
Other genes ubicated in the mitochondria membrane	*RDL1, ISC1*	*COQ8, YGR266W, OMS1*	*ZEO1, OM45, RDL1*	*YBR238C, UTH1*
Protein modification		*MAS2*	*PTH2, ISA2*	*YTA12, UBX2, PTC5, YME1*
Other	*ISU1, MMF1, VPS73*	*CIR2, UBP16, PUF3, AIF1, AIM36, MIX17, POS5, DRE25*	*YBL059W, YPT7, FYV4, ISU2, YSC83, CIR1, AIM19, VPS1, YCR028C-A, ATG9, FMC1, YSP2, MIX14, FMP33, HMF1, ECM10*	*PKP1, CAF4*

## Supplementary Materials

The following supplementary materials are available online at https://www.mdpi.com/2076-3921/9/10/947/s1, Figure S1: Genetic interactions analyzed by Phenetics (A) Mel vs. Control. (B) MelH vs. H; Table S1: Categories enriched according to GO analysis of the common genes identified in different comparisons, as shown in a Venn diagram (Figure 1); Table S2: Enriched pathways according to a KEGG database analysis of genes that were differentially expressed among cells in Mel vs. Control and in MelH vs. H; Table S3: Statistical results of the ANOVA test for the data in Figure 5. Table S4: Gene expression values representing the effect of melatonin on the nine selected genes in Mel vs. Control and MelH vs. H cells; Datasets S1–S10: Microarray analysis data of the different comparisons, statistics (moderated t-test) and annotations for each gene. Genes filtered with *p*-value < 0.05 (S1–S5) or no filtered by *p*-value (S6-S10): S1,S6: Mel vs Control; S2,S7: H vs. Control, S3,S8: MelH vs. Control; S4,S9: MelH vs. Control; S5,S10: 25MelH vs. MelH; Datasets S11–S19: Results of the GO enrichments [molecular function (11, 14, 17), cellular component (12, 15, 18) and biological process (13, 16, 19)] of the genes differentially expressed in Mel vs. Control (11–13), MelH vs. H (14–16), H vs. Control (17–19).
